# Person–Job Fit and Innovation Behavior: Roles of Job Involvement and Career Commitment

**DOI:** 10.3389/fpsyg.2019.01134

**Published:** 2019-05-16

**Authors:** Wenyuan Huang, Chuqin Yuan, Min Li

**Affiliations:** School of Business Administration, South China University of Technology, Guangzhou, China

**Keywords:** person–job fit, job involvement, innovation behavior, career commitment, person–environment fit theory, identity theory

## Abstract

This study examines the effect of person–job fit on innovation behavior, highlighting the mediating role of job involvement and the moderating role of career commitment in this relationship. We tested our hypotheses using a sample of 474 employees from 30 IT enterprises in China’s Pearl River Delta region. The results reveal that person–job fit influences innovation behavior by enhancing job involvement. In addition, career commitment strengthens the positive influence of person–job fit on both job involvement and innovation behavior. These findings are consistent with person–environment fit theory and identity theory. This research increases understanding of how person–job fit influences innovation behavior via job involvement and career commitment. Implications and managerial practice are also discussed at the end of the research.

## Introduction

With the rapid development of technology and economic globalization, organizations face not only a complex and changeable external environment but also need to satisfy customers and stakeholders’ diverse demands (Madrid et al., 2014). Therefore, modern organizations cannot solely depend on traditional or standard rules and procedures to guarantee success (Janssen, 2000). Instead, actions oriented toward effectively managing unforeseen work situations or exploiting new opportunities in the workplace are essential for achieving success (Kanter, 1988; West and Farr, 1990). In this context, employee innovation behavior, which is regarded as a resource of organizational innovation (Amabile et al., 1996; Ding et al., 2018), has attracted significant scholarly attention (e.g., Madrid et al., 2014; Mussner et al., 2017; Che et al., 2018; Kim et al., 2018).

Since employee innovation behavior has long been regarded as a significant determinant of organizational performance, competitive advantage, and long-term survival, many researchers have investigated how to foster it (Tu and Lu, 2013; Madrid et al., 2014; Afsar et al., 2015). These studies have generally focused on examining innovation behavior’s antecedents in an organizational context; among those identified are organizational justice, job characteristics, the psychological contract, intrinsic motivation, rewards, leadership, and working relationship quality (e.g., Scott and Bruce, 1994; Dorenbosch et al., 2005; Ramamoorthy et al., 2005; Reuvers et al., 2008; De Jong and Den Hartog, 2010). However, they are limited by focusing on either individual or job characteristics, rather than both. This is unhelpful for predicting employees’ innovation behavior, which often results from communication, friction, and interaction between individuals and their environment (Ashforth et al., 2007).

With more in-depth research on the topic of person–environment fit, attention has been gradually given to the effect of person–job fit, which emphasizes the match between individual knowledge, skills, abilities, and job requirements (Zhou et al., 2011). Numerous studies report that an employee whose personal values fit better with the values of their job description show higher levels of citizenship behavior (e.g., Goodman and Svyantek, 1999; Vigoda, 2000; Kristof-Brown et al., 2005; Farzaneh et al., 2014). Since innovation behavior is a purely discretionary citizenship behavior, not mandated in formal job descriptions and roles (Janssen, 2000; Ramamoorthy et al., 2005), the effect of person–job fit on innovation behavior warrants exploration. Unfortunately, few empirical studies have explored the influence mechanism of person–job fit on innovation behavior (Zhao and Han, 2016). Afsar et al. (2015) and Lin and Ding (2017), respectively, adopted the perspective of innovative self-efficacy and innovation trust to explore the link between person–job fit and innovation behavior, indicating that there are multiple interpretations of this relationship. Their research also provides a reference for us to consider the mediating role in the relationship of job involvement, which has been considered as the key to activating employee motivation (Lawler, 1986) and an important mechanism for transforming inducing factors into the employee attitudes and behaviors expected by organizations (e.g., Shantz et al., 2016; Welbourne and Sariol, 2017; Ćulibrk et al., 2018; Liu and Gu, 2018). Zhao and Han (2016) contend that employees who fit well with their job requirements have enough resources to devote to their work, which enhances work motivation and increases job involvement. Furthermore, where such employees are involved in the creative process, they can more effectively identify problems or challenges and gather information, before then proposing more efficient solutions (Kim et al., 2010). However, scholars have not yet theoretically modeled the relationship between person–job fit, job involvement, and innovation behavior. Accordingly, this study’s first aim is to test the mediating role of job involvement between person–job fit and innovation behavior.

Zhang and Long’s (2013) empirical study found that the relationship between person–job fit and its outcomes is influenced by individual factors and results in an incomplete conclusion if we neglect such contingency factors. According to identity theory (Stets and Serpe, 2013), employees with low levels of career commitment can be expected to display low levels of job involvement and innovation behavior because they tend to exhibit less positive work attitudes and behavior in general (Duffy et al., 2011; Pei and Zhao, 2015), which is not conducive to optimizing organizational performance. Therefore, the role of career commitment must be taken seriously. Blau (2009) contends that career commitment is becoming a better predictor of employees’ attitudes and behaviors because it reflects behavioral choices of employees in today’s unstable labor force. In particular, with increased levels of education, flexibility, and mobility among employees, the organization is no longer an employee’s only commitment in the workplace; rather, other forms of commitment, such as career commitment, are becoming increasingly important (Cohen, 2011). Therefore, this study’s second aim is to test career commitment as a moderator in the relationship of person–job fit with job involvement and innovation behavior.

Our study contributes to the literature by considering job involvement as an additional explanatory mechanism in the relationship between person–job fit and innovation behavior, which supplements and adds a new perspective to the explanation of the relationship between person–job fit and innovation behavior. Furthermore, drawing from identity theory, we theorize and examine career commitment as a moderator between person–job fit and both job involvement and innovation behavior, thereby deepening understanding of the contingency factors that influence the outcomes of person–job fit.

## Theoretical Background and Hypotheses Development

### Person–Job Fit and Innovation Behavior

Person–job fit is defined as the degree of alignment between the individual and the job (Wong and Tetrick, 2017). Previous studies indicate that person–job fit is positively related to work engagement (Cai et al., 2018) and contextual performance (Han et al., 2015), but negatively related to employee turnover (Boon and Biron, 2016). Person–job fit is generally regarded as a positive element in the workplace. Regarding the relationship between person–job fit and employee innovation behavior, Amabile (1988) asserts that an individual’s knowledge and skills in a certain field are key to their creative performance and action in that field. In the classical interaction model of creativity proposed by Woodman et al. (1993), ability and knowledge are two important antecedents of individual creativity. Ford’s (1996) creative action model also emphasizes that knowledge and ability in a certain field are important driving factors for individual creative action. Amabile (1988) contends that domain-related expertise, creativity skills, and task motivation are the three main factors that influence creativity. She also asserts that domain-related expertise is the foundation of all creative work and comprises a set of cognitive pathways that can be followed to solve a given problem or accomplish a specific task. On this basis, the higher the match between an employee and their job, in terms of position-related knowledge and skills, the higher should be the resulting level of employee innovation behavior. In addition, employees whose skill set strongly matches their job description are considered to have sufficient comprehension and abilities to meet their job requirements and a stronger aptitude for managing the innovation process (Zhao and Han, 2016). According to Ajzen and Fishbein (1977), employees who believe they have strong control in the innovation process will manifest strong willingness to innovate and demonstrate more innovation behaviors. Based on the above arguments, we hypothesize:

*Hypothesis 1 (H1)*: Person–job fit is positively related to innovation behavior.

### The Mediating Role of Job Involvement

In recent years, person–environment fit theory has attracted increasing scholarly attention. It holds that individual behavior is the function of the interaction between individual and environment, wherein a good match between an employee and their organizational environment can produce positive employee attitudes and behaviors. A strong match between an employee and their job description means that they possess adequate resources to devote to their work (Zhao and Han, 2016), and highly involved employees are more likely to engage in innovation behavior (Gu et al., 2014). Moreover, available job resources can drive innovation by enabling employees to devote more enthusiasm and energy to effectively responding to job demands from which they obtain personal benefits and growth (Sun et al., 2018). Therefore, we speculate that job involvement may mediate the relationship between person–job fit and innovation behavior.

Job involvement refers to the degree of employee’s psychological identification with the job (Kanungo, 1982). It is not only an important source of individual performance improvement (Park et al., 2010) but also a key factor for an organization to maintain competitive advantage (Ebeh et al., 2017). Increasing employees’ involvement in their work can foster a sense of value that may motivate greater devotion to workplace tasks. Kanungo (1982) argues that the extent to which employees’ psychological needs are met by their job is an important determinant of the degree of job involvement; therefore, the more the job characteristics and work situation meet employees’ psychological needs, the more they identify with and become involved in their work. Employees strongly matched with their jobs possess the knowledge and skills necessary to fulfill their tasks, leading to recognition and respect from leaders, self-organizing support, and more workplace autonomy. Their basic psychological needs are also satisfied (Zhao and Han, 2016). Thus, person–job fit is positively related to job involvement.

For employees with high job involvement, their jobs seem inexorably aligned with their interests, identities, and life goals, and are important (Mudrack, 2004). Job involvement develops in employees through a long and meaningful process (Taştan, 2013). The popular research assumption is that job involvement is an innate quality of employees (Mudrack, 2004), since employees with high job involvement tend focus on their work and devote “personal resources” to their current position (Kanungo, 1982). In fact, employees who are highly job-involved are more likely to be satisfied with their jobs, exhibit positive moods in the workplace, and be committed to their current organizations, careers, and professions (Carson et al., 1995; Cohen, 1995). In addition, employees with high job involvement perceive harmony between their personal and organizational goals (Chay and Aryee, 1999); are inclined to focus on job activities even in leisure time, such as seeking out ways to further enhance their performance (Mudrack, 2004); and feel competent and successful, and assist co-workers in generating innovation in the organization (Holton and Russell, 1997; Dimitriades, 2007). Finally, empirical studies have already demonstrated a positive relationship between job involvement and innovation behavior (Hoffi-Hofstetter and Mannheim, 1999; Sarros et al., 2011). Based on the above arguments, we hypothesize:

*Hypothesis 2 (H2):* Job involvement mediates the relationship between person–job fit and innovation behavior.

### The Moderating Role of Career Commitment

Career commitment refers to employee satisfaction with their current career and their desire to continue therein, focusing mainly on emotional components (Blau, 1985). As Colarelli and Bishop (1990) intimate, career-committed employees will set career goals and then identify with and be involved in those endeavors. Conversely, employees with low career commitment are likely to have less job satisfaction and organizational commitment (Duffy et al., 2011; Pei and Zhao, 2015), which is not conducive to achieving meaningful individual performance.

Lee et al. (2000) contend that employees with high career commitment are more willing to engage in work and achieve higher job performance than those with low career commitment. Although person–job fit can positively influence employee attitude and behavior, this process may be inhibited if employees do not identify with their current occupations. By contrast, if employees are highly committed to their current occupation, they are more likely to expend time and resources developing their skills and are less willing to abandon their careers (Aryee and Tan, 1992); in turn, job involvement and innovation behaviors are fostered. In addition, employees with high career commitment will seek to understand the needs of the organization, and make proactive adjustments to align their personal goals with organizational goals (Wang et al., 2017), which is also conducive to generating job involvement and innovation behaviors. Chang (1999) found that employees with high career commitment had stronger motivation (compared to low-career-commitment employees) when their expectations were supported and met by the organization. In line with the above discussion, we hypothesize:

*Hypothesis 3 (H3):* Career commitment positively moderates the effect of person–job fit on job involvement—the relationship is stronger when career commitment is high (rather than low).
*Hypothesis 4 (H4):* Career commitment positively moderates the effect of person–job fit on innovation behavior—the relationship is stronger when career commitment is high (rather than low).
*Hypothesis 5 (H5):* Career commitment positively moderates the mediating effect of job involvement between person–job fit and innovation behavior—the mediating effect is stronger when career commitment is high (rather than low).

**FIGURE 1:**
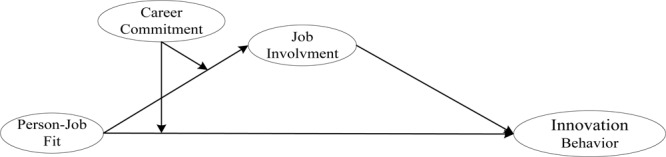
Theoretical model.

The study’s theoretical model is depicted in Figure 1.

## Materials and Methods

### Sample and Procedure

To collect data for this study, 600 questionnaires were distributed among 30 IT companies in China’s Pearl River Delta region between July and August 2017. The researchers first contacted each company’s human resources (HR) manager to request their assistance with data collection. After obtaining HR managers’ consent, the researchers mailed copies of the printed questionnaire to them with a request to organize questionnaire completion by staff. Each participating employee placed their completed questionnaire in a sealed envelope and handed it directly to their HR manager. Finally, HR managers mailed the (still-sealed) completed questionnaires back to the researchers. To ensure data authenticity and accuracy, assurance of anonymity and confidentiality was given to respondents on the front page of the questionnaire, together with a brief outline of how responses would be used.

In total, 526 questionnaires were returned, of which 474 were used for analyses after elimination of invalid questionnaires. Regarding the sample’s demographics, 53.4% were male and 46.6% female; the average age was 27.83 years (*SD* = 4.45); the average organizational tenure was 2.4 years (*SD* = 2.08); and 67.1% had completed college and/or postgraduate education.

### Measures

This study employed widely-used measures compiled by Chinese and other scholars. Except for the person–job fit scales, job involvement, innovation behavior, and career commitment were developed in English and were showed in Chinese. All the items in each measure were scored on a five-point Likert scale, ranging from 1 = strongly disagree to 5 = strongly agree.

### Person–Job Fit

Person–job fit was measured using a 4-item questionnaire developed and validated by Weng (2010). A sample item is “The requirements of my job match my specific talents and skills.” Cronbach’s alpha for this scale was 0.93.

### Job Involvement

Job involvement was measured using a 3-item questionnaire developed and validated by Reilly et al. (1993). A sample item is “The most important things that happen to me involve my work.” Cronbach’s alpha for this scale was 0.73.

### Innovation Behavior

Innovation behavior was measured using a 6-item questionnaire developed and validated by Scott and Bruce (1994). A sample item is “I search out new technologies, processes, techniques, and product ideas.” Scott and Bruce’s (1994) innovation measure was found to be correlated with objective measures of innovation behavior (Eva et al., 2017). It has also been used for both self-reported and manager-reported innovation (Tu and Lu, 2013). We chose the former for the three reasons outlined by Janssen (2000). First, superiors may not be present for or privy to many of the innovation behaviors of employees during their daily tasks. Second, employees have greater understanding of the historical and contextual backgrounds for their tasks, and so are more cognitively aware of subtle changes in their tasks. Third, innovation behaviors, like many other work behaviors, are highly susceptible to personal biases and can differ across raters. Self-report measures have been commonly used in organizational behavior research (Ng et al., 2010), and previous studies show that self-reported and manager-rated innovation (*r* = 0.35, *p* < 0.01) has a significant correlation (Janssen, 2000). Cronbach’s alpha for this scale was 0.92.

### Career Commitment

Career commitment was measured using an 8-item questionnaire developed and validated by Blau (1985). A sample item is “I like this profession too much to give it up.” Cronbach’s alpha for this scale was 0.93.

### Control Variables

In line with previous research (e.g., Mumford et al., 2002; Jung et al., 2003; Tu and Lu, 2013; Madrid et al., 2014), several control variables such as gender, age, and organizational tenure were also included at the individual level. We also identified education level as a control variable.

## Results

### Minimization of Common Method Variable

Although we reminded participants of the anonymity of their questionnaire responses during the collection process, all the data are single source. This is significant with respect to potential common method variance. As Harman’s single factor test has been criticized as “insensitive” (Fuller et al., 2016), we employed partial least squares (PLS) to test for common method variance in this study (Liang et al., 2007). As Table 1 shows, all indicators loading on the proposed latent variable are not smaller than 0.50 and their loadings on the common method latent variable (CMLV) are all non-significant. The results clearly indicate that common method variance is not a problem for the data in this study (Williams et al., 2003; Brammer et al., 2015).

**Table 1:** PLS analysis.

Construct	Indicator	Loading to proposed latent variables	Loading to CMLV
Person–job fit	PJ1	0.91***	–0.15
	PJ2	0.89***	–0.12
	PJ3	0.80***	–0.33
	PJ4	0.87***	–0.12
Career commitment	CC1	0.72***	–0.26
	CC2	0.82***	–0.24
	CC3	0.81***	–0.32
	CC4	0.83***	–0.35
	CC5	0.86***	–0.08
	CC6	0.84***	0.29
	CC7	0.84***	0.14
	CC8	0.69***	0.16
Job involvement	JI1	0.73***	–0.29
	JI2	0.79***	–0.33
	JI3	0.58***	0.16
Innovation behavior	IB1	0.81***	0.06
	IB2	0.82***	0.08
	IB3	0.86***	–0.03
	IB4	0.80***	–0.11
	IB5	0.80***	–0.15
	IB6	0.78***	0.24
Average	–	0.80	–0.08

*^∗∗∗^*p* < 0.001.*

### Confirmatory Factor Analysis

A series of confirmatory factor analyses were used to evaluate the discriminant validity among variables in the study. As Table 2 shows, the four-factor model has acceptable fitness, whereas the one-factor model, two-factor model, and three-factor model were unacceptable. This demonstrates that the four measures were empirically distinct from one another (Harris and Mossholder, 1996).

**Table 2:** Comparison of measurement models.

Models	χ^2^	*df*	RMSEA	SRMR	CFI	TLI
Four-factor model	1169.49	183	0.11	0.05	0.87	0.86
Three-factor model	1576.07	186	0.10	0.12	0.82	0.80
Two-factor model	2957.45	188	0.18	0.15	0.65	0.61
One-factor model	4151.18	189	0.21	0.16	0.49	0.44

*One-factor model, all items were loaded on one factor. Two-factor model, person–job fit, job involvement, innovation behavior and career commitment were loaded on one factor. Three-factor model, person–job fit and job involvement were loaded on one factor, career commitment, innovation behavior. Four-factor model, person–job fit, job involvement, innovation behavior, career commitment.*

### Descriptive Statistics

Table 3 reports the means, standard deviations, and correlation among the variables. The results indicate that the data could be tested for mediation and moderation.

**Table 3:** Means, standard deviations, and correlations.

	*M*	*SD*	1	2	3	4	5	6	7	8
1. Gen	1.46	0.50								
2. Age	27.83	4.45	–0.09*	–						
3. Edu	2.73	0.62	–0.13**	0.22**	–					
4. OT	2.40	2.08	–0.01	0.44**	0.08	–				
5. P–J fit	3.55	0.79	–0.11*	0.14**	0.01	0.02	–			
6. CC	3.60	0.73	–0.06	0.19**	0.02	0.09*	0.48**	–		
7. JI	3.44	0.84	0.01	0.06	–0.13**	0.04	0.29**	0.43**	–	
8. IB	3.44	0.72	–0.14**	0.12**	0.06	–0.03	0.35**	0.38**	0.34**	–

*^∗^*p* < 0.05, ^∗∗^*p* < 0.01, ^∗∗∗^*p* < 0.001. OT, organizational tenure; P–J fit, person–job fit; CC, career commitment; JI, job involvement; IB, innovation behavior.*

### Main and Mediation Effects Test

Structural equation modeling and a bootstrap approach were performed in Mplus 7.4 to test H1 and H2, and the results are shown in Table 4. Person–job fit was positively related to innovation behavior (γ = 0.25, *p* < 0.01), thus H1 was supported. To test the indirect effect of person–job fit on innovation behavior through job involvement, we used 2,000 bootstrapping samples and then reported bias-corrected confidence intervals. The indirect effect was found to be significant (γ = 0.10, *SD* = 0.02, 95%CI = [0.07, 0.15]), so H2 was supported.

**Table 4:** Results of bootstrap.

Variables	Job involvement		Innovation behavior	
	Value	*SD*	Value	*SD*
Gen	–0.11	0.10	–0.17	0.08
Age	0.00	0.01	0.02	0.01
Edu	–0.22	0.08	0.12	0.06
OT	–0.01	0.02	–0.03	0.02
P–J fit	0.38^∗∗^	0.07	0.25^∗∗^	0.06
JI			0.28^∗∗^	0.05

*N = 474, ^∗^*p* < 0.05, ^∗∗^*p* < 0.01; OT, organizational tenure; P–J fit, person–job fit; JI, job involvement.*

### Moderation Effect Test

**FIGURE 2:**
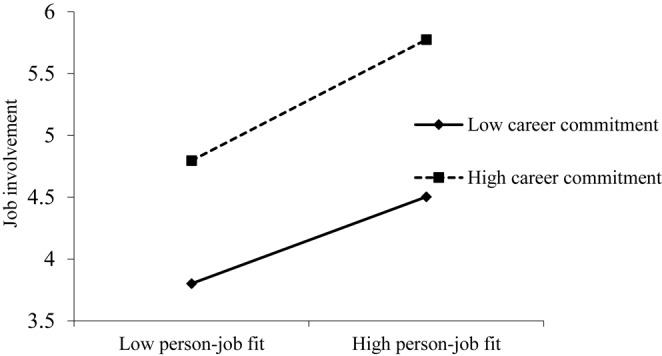
Interactive effect of person–job fit and career commitment on job involvement.

**FIGURE 3:**
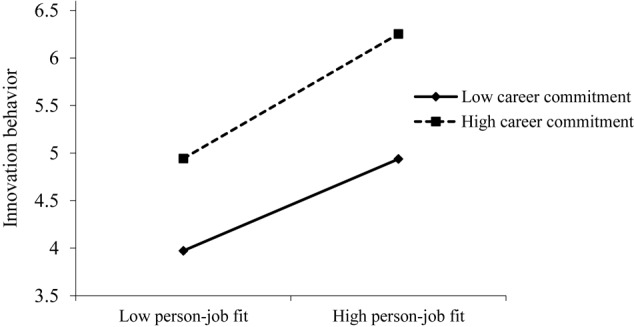
Interactive effect of person–job fit and career commitment on innovation behavior.

To test the moderation effect of career commitment between person–job fit and both job involvement and innovation behavior, ordinary least squares regression analysis was conducted in SPSS.24.0. As Table 5 shows, the interaction of person–job fit and career commitment was significant in predicting job involvement (γ = 0.12, *p* < 0.01, M2) and innovation behavior (γ = 0.15, *p* < 0.01, M4). Further, the relationship between person–job fit and job involvement (Figure 2) was stronger for employees with high career commitment than for those with low career commitment. Moreover, the relationship between person–job fit and innovation behavior (Figure 3) was stronger for employees with a high career commitment than for those with low career commitment. Thus, H3 and H4 are both supported.

**Table 5:** Moderation effects.

Variables	Job involvement		Innovation behavior	
	M1	M2	M3	M4
Gen	–0.05	0.02	–0.12	–0.09^∗^
Age	0.08	–0.02	0.15^∗∗^	0.05
Edu	–0.15^∗∗^	–0.12^∗∗^	0.02	0.06
OT	0.02	0.02	–0.10	–0.09
P–J fit		0.10^∗^		0.18^∗∗^
CC		0.35^∗∗^		0.25^∗∗^
P–J fit^∗^CC		0.12^∗∗^		0.15^∗∗^
*R*^2^	0.02	0.22	0.04	0.22
*F*	2.82^∗^	18.87^∗∗^	4.85^∗∗^	18.59^∗∗^
Δ*R*^2^	0.02	0.20	0.04	0.18
Δ*F*	2.82^∗^	39.34^∗∗^	4.85^∗∗^	35.48^∗∗^

*N = 474, ^∗^*p* < 0.05, ^∗∗^*p* < 0.01; OT, organizational tenure; P–J fit, person–job fit; CC, career commitment.*

### Moderated Mediation Test

We tested H5 using the moderated path analysis approach with 2,000 bootstrapping samples (Edwards and Lambert, 2007) and Mplus7.4 software. The results are reported in Table 6. The indirect impact coefficient of job involvement between person–job fit and innovation behavior is significant for both high career commitment (γ = 0.18, 95% CI = [0.06, 0.32]) and low career commitment (γ = 0.13, 95% CI = [0.04, 0.22]), which again supports H3. The difference in the impact coefficient of job involvement between person–job fit and innovation behavior is also significant (γ = 0.05, 95% CI = [0.02, 0.11]). Thus, H5 was supported.

**Table 6:** Results of the moderated mediation.

Mediating path	Moderator variable	Coefficient		95% CI	
		Effect	*SD*	Lower 2.5%	Upper 2.5%
Person–job fit→	High career commitment	0.18	0.08	0.06	0.32
job involvement→	Low career commitment	0.13	0.05	0.04	0.22
innovation behavior	Difference	0.05	0.03	0.02	0.11

## Discussion

Based on person–environment fit theory and identity theory, we built and tested a model to explore the influence mechanism of person–job fit on innovation behavior, focusing on the mediating role of job involvement and the moderating role of career commitment. The results show that: (1) person–job fit promotes innovation behavior by stimulating the employee’s job involvement; (2) career commitment strengthens the positive impact of person–job fit on job involvement and innovation behavior; and (3) career commitment strengthens the positive impact of person–job fit on job involvement and further promotes the formation of innovation behavior. These findings have important implications for the development of relevant theories and the practice of enterprise innovation management.

### Theoretical Implications

First, job involvement plays a mediating role between person–job fit and innovation behavior. Whether and how person–job fit influences innovation behavior has been a hot topic since Woodman et al. (1993) proposed the interaction model of creativity. As an important component of person–environment, person–job fit has been proved to have a significant predictive effect on employee innovation behavior. However, the influence mechanism of person–job fit on innovation behavior has attracted less scholarly attention. Although previous studies have proved the mediating role of perceived insider status, abusive supervision, and innovation trust between person–job fit and innovation behavior (Afsar et al., 2015; Zhao and Han, 2016; Lin and Ding, 2017), the mediating role of job involvement in this relationship has not been thoroughly studied. According to Kim et al. (2010), employees who devote more time and energy to the creative process can more effectively identify problems, collect information, and propose solutions to challenges. Therefore, we analyzed the influence mechanism of person–job fit on innovation behavior from the perspective of job involvement, based on person–environment fit theory. Our study confirms the positive effect of person–job fit on innovation behavior and also reveals the mediating role between them of job involvement. These findings not only enrich person–environment fit theory but also complement and expand the literature on innovation behavior. In addition, our findings are helpful for understanding the relationship between person–job fit and innovation behavior from a different perspective, while also providing new ideas for studying the influence mechanism of person–job fit on innovation behavior.

Second, this study identifies the reinforcement conditions of person–job fit on both job involvement and innovation behavior. It also confirms that personal resources may influence individual reactions to job characteristics (Wu et al., 2010; Bakker and Demerouti, 2017). The moderating role of career commitment indicates situational constraints on the extent to which employee job involvement and innovation behavior can be nurtured and stimulated. The conclusions provide a theoretical basis for opening the “black box” between person–job fit and innovation behavior, thus offering clearer understanding of the boundaries of the influence of person–job fit. At the same time, it is evidently necessary for follow-up research on the relationship between person–job fit and its outcomes to consider the boundary conditions, such as perceived insider status (Lin and Ding, 2017) and job insecurity (Zhang and Long, 2013).

### Practical Implications

This study’s findings also have important managerial implications. First, emphasize the best match between employees and their tasks and responsibilities has long been the goal of organizations’ HR managers. For this reason, significant investment is made in human and financial resources, such as strict recruitment and selection, thorough and specific training, and different forms of deployment and promotion measures. Although theoretical studies have confirmed that person–job fit has a significant positive effect on employee outcome variables such as job performance and organizational commitment (Kristof-Brown et al., 2005), this has not previously been supported by systematic research on whether such costly investment promotes greater creative performance by employees. This study’s results confirm that person–job fit has a significant positive effect on innovation behavior, which indicates the value of investment based on person–job fit because it is conducive to improving employee creative performance.

Second, person–job fit contributes to promoting employee job involvement, which in turn promotes innovation behavior. Therefore, HR managers should improve the degree of fit between employees and their jobs through streamlining the selection process, recruitment, pre-job training, and other proactive methods to guarantee employees actively involved in work to enhance creative performance.

Finally, organizational directors and supervisors should pay greater attention to their employees’ career development planning and to whether its direction is consistent with employees’ current positions. In addition, managers and supervisors need to consider employee career commitment levels. For employees with low career commitment, it would be advantageous for managers to provide vocational training or ask career planners to offer guidance, so as to clarify employees’ career orientation and enhance their focus on the profession.

### Limitations and Future Directions

Although several important conclusions can be derived from this study, three main limitations must be considered when interpreting the results. First, cross-sectional data were used to test the theoretical model, which limited our ability to establish a causal relationship between independent and dependent variables. We recommend use of longitudinal data in future research to examine how person–job fit leads to innovation behavior. A second limitation is that all our respondents were employees of enterprises in China’s Pearl River Delta region, which may influence our findings’ generalizability to other areas and cultural contexts. Therefore, the conclusions of our findings need more replication researches to ensure its theoretical implications. Finally, our research only considers one moderator variable (career commitment); future research should explore other moderator variables such as perceived insider status, organizational commitment, or person-organization fit in the innovation behavior process.

## Ethics Statement

This study was carried out in accordance with the recommendations of the Ethics Committee of South China University of Technology. The protocol was approved by Ethics Committee of South China University of Technology. All subjects gave written informed consent in accordance with the Declaration of Helsinki.

## Author Contributions

WH contributed to developing the theoretical framework, data analysis organization, and overall writing of the manuscript. ML contributed to data collection and the editing and organization of the manuscript. CY has read the manuscript and approved its definitive version.

## Conflict of Interest Statement

The authors declare that the research was conducted in the absence of any commercial or financial relationships that could be construed as a potential conflict of interest.
